# Heavy Metal Pollution Impacts Soil Bacterial Community Structure and Antimicrobial Resistance at the Birmingham 35th Avenue Superfund Site

**DOI:** 10.1128/spectrum.02426-22

**Published:** 2023-03-23

**Authors:** Anuradha Goswami, Sarah J. Adkins-Jablonsky, Marcelo Malisano Barreto Filho, Michelle D. Shilling, Alex Dawson, Sabrina Heiser, Aisha O’Connor, Melissa Walker, Qutia Roberts, J. Jeffrey Morris

**Affiliations:** a Department of Biology, University of Alabama at Birmingham, Birmingham, Alabama, USA; b Alabama College of Osteopathic Medicine, Dothan, Alabama, USA; University of Texas at San Antonio

**Keywords:** antimicrobial resistance, topsoil, heavy metal pollution, superfund site

## Abstract

Heavy metals (HMs) are known to modify bacterial communities both in the laboratory and *in situ*. Consequently, soils in HM-contaminated sites such as the U.S. Environmental Protection Agency (EPA) Superfund sites are predicted to have altered ecosystem functioning, with potential ramifications for the health of organisms, including humans, that live nearby. Further, several studies have shown that heavy metal-resistant (HMR) bacteria often also display antimicrobial resistance (AMR), and therefore HM-contaminated soils could potentially act as reservoirs that could disseminate AMR genes into human-associated pathogenic bacteria. To explore this possibility, topsoil samples were collected from six public locations in the zip code 35207 (the home of the North Birmingham 35th Avenue Superfund Site) and in six public areas in the neighboring zip code, 35214. 35027 soils had significantly elevated levels of the HMs As, Mn, Pb, and Zn, and sequencing of the V4 region of the bacterial 16S rRNA gene revealed that elevated HM concentrations correlated with reduced microbial diversity and altered community structure. While there was no difference between zip codes in the proportion of total culturable HMR bacteria, bacterial isolates with HMR almost always also exhibited AMR. Metagenomes inferred using PICRUSt2 also predicted significantly higher mean relative frequencies in 35207 for several AMR genes related to both specific and broad-spectrum AMR phenotypes. Together, these results support the hypothesis that chronic HM pollution alters the soil bacterial community structure in ecologically meaningful ways and may also select for bacteria with increased potential to contribute to AMR in human disease.

**IMPORTANCE** Heavy metals cross-select for antimicrobial resistance in laboratory experiments, but few studies have documented this effect in polluted soils. Moreover, despite decades of awareness of heavy metal contamination at the EPA Superfund site in North Birmingham, Alabama, this is the first analysis of the impact of this pollution on the soil microbiome. Specifically, this work advances the understanding of the relationship between heavy metals, microbial diversity, and patterns of antibiotic resistance in North Birmingham soils. Our results suggest that polluted soils carry a risk of increased exposure to antibiotic-resistant infections in addition to the direct health consequences of heavy metals. Our work provides important information relevant to both political and scientific efforts to advance environmental justice for the communities that call Superfund neighborhoods home.

## INTRODUCTION

Heavy metals (HMs) are necessary for biological processes across all domains of life (e.g., by acting as catalytic cofactors in proteins), but they can also be toxic in high concentrations. HMs in soil environments are documented human health risks (1–3), a fact all too familiar to people in the community of North Birmingham, Alabama. For decades, environmental injustice in the form of industrial pollution from coke furnaces and steel plants has plagued residents living in this Central Alabama area, over 90% of whom are African-American and 40% of whom live under the federal poverty line (4). These facilities emit particulate matter containing HMs such as Fe, Zn, Pb, Cu, Cr, Cd, As, and Mn into the air and soil (5, 6). In recognition of the potential health impacts caused by this large-scale pollution, the Environmental Protection Agency (EPA) designated North Birmingham as the 35th Avenue Superfund Site in 2012 (henceforth referred to by its zip code, 35207), committing the U.S. federal government to fund pollution cleanup (4). However, continued pollution and local politics have stalled the EPA’s progress, and 35207 residents have yet to see substantial progress toward confronting and overcoming the legacy of environmental mismanagement. Grassroots organizations such as People Against Neighborhood Industrial Contamination (PANIC) have asked for remediation and financial restitution for 35207 residents but also more scientific studies to understand and quantify the environmental and health impacts explicitly related to HM contamination in their community (7).

Chronic HM exposure is known to have several effects on microbial communities. HM pollution can select for specific bacterial taxa and physiological properties (8), which can in turn impact the diversity of the microbial populations (8–10). For instance, HMs can affect soil properties, such as spatial structure, in ways that in turn increase bacterial community diversity (11, 12). HMs are also known to cross-select for both heavy metal resistance (HMR) and broad antibiotic/antimicrobial resistance (AMR) in bacteria (13–16), even at low levels (17), because many of the mechanisms conferring resistance to one set of toxins are also effective at resisting the other as well. Laboratory experiments with bacterial cultures showed, for example, that Cd exposure induced transmembrane efflux pump resistance not only to HMs such as Zn and Cd, but also to carbapenem antibiotics (18).

Thus, it is possible that 35207 soils contain microbial communities not only with different microbial taxa than surrounding soils, but perhaps also with high levels of AMR genes. The development of bacterial cross-resistance via HMR and/or AMR genes can occur through horizontal gene transfer (19–22), and therefore it is possible that these bacteria could function as a reservoir from which AMR could spread to the human-associated bacteria of 35207 residents, creating an additional health risk beyond the direct impacts of HM exposure. Alarmingly, many AMR genes confer broad resistance across antibiotic classes (23). According to the World Health Organization (24), the spread of AMR is one of the most pressing concerns for the 21st century. Given that 35207 residents are already more vulnerable to infectious diseases (25) and that AMR infections continue to rise globally (26), investigating the potential for industrial HM pollution to cause AMR emergence at Superfund sites such as 35207 is a social justice imperative. To investigate this possibility, we collected topsoil samples from 35207 as well as from a nearby Birmingham neighborhood with similar demographics but farther from the EPA-designated Superfund site (henceforth also referred to by its zip code, 35214) and used them to address the following questions:

1. Do the soil bacterial communities at the North Birmingham Superfund site differ from those in an adjacent neighborhood with less pollution?
2. Do HM-polluted regions show greater physiological or predictive genetic evidence of AMR?

## RESULTS

### Impact of HM pollution on soil bacterial community structure.

**FIG 1:**
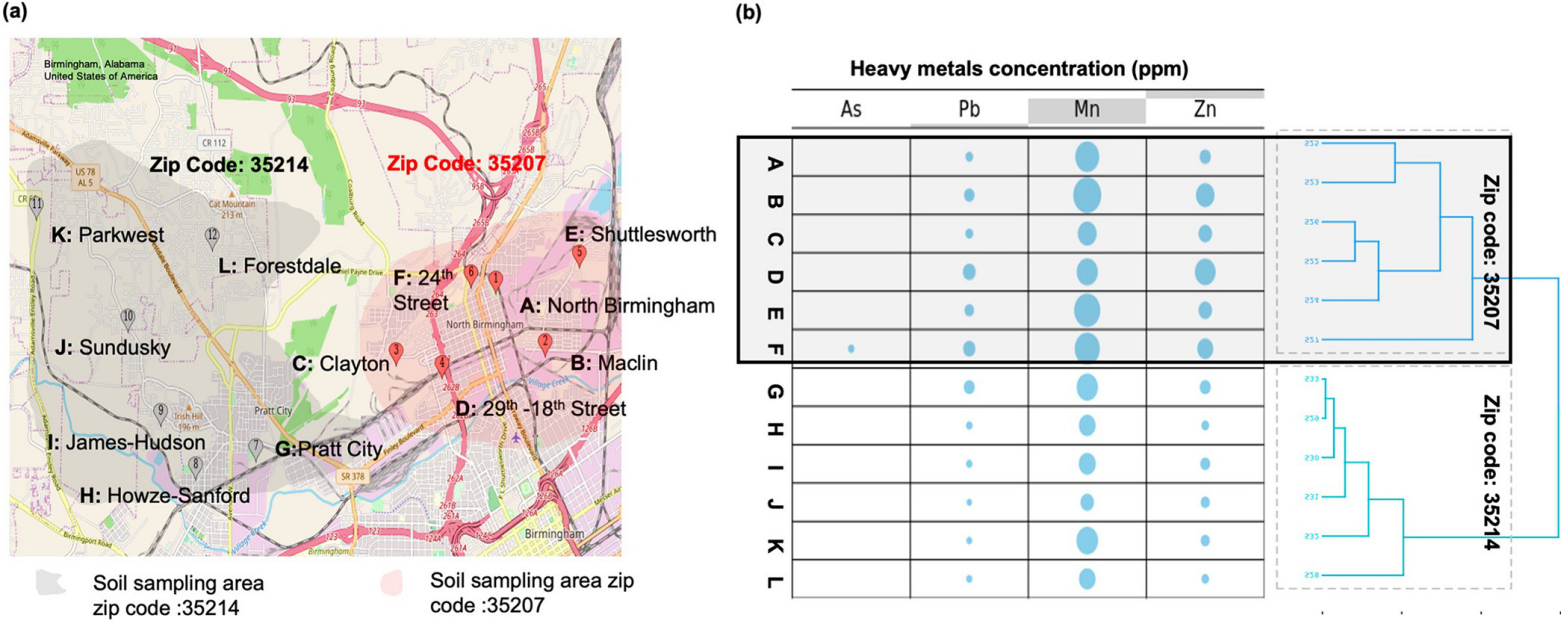
(a) Map of sampling sites A to F from 32507 (in red) and G to K from 35214 (in gray) (map generated by GoogleMaps Map Customizer with longitude and latitude points is accessible at https://www.mapcustomizer.com/map/Nbham%20paper). (b) Relative HM concentrations are depicted in the bubble chart, where larger bubble size corresponds to larger heavy metal concentrations (range 26 to 822ppm). Metal analytes with no bubble indicate a concentration below the detection limit as described in Materials and Methods. The dendrogram on the right of the chart shows the kmer clustering of sampling sites based on HM concentrations.

Soils from sampling sites in 35207, which contains the Superfund site, had significantly higher levels of Pb, Mn, and Zn than those in 35214, a comparable neighborhood farther away from pollution sources (Fig. 1; Mann-Whitney U tests, *P* < 0.05). As and Cd were below detection limits for all samples except for 35207 site F, having 37 ppm As. Average silhouette width kmer clustering based on metal concentrations supported clustering the samples into two groups that corresponded exactly to the two neighborhood zip codes, supporting our hypothesis that 35207 soils were significantly more exposed to HM than 35214 soils (Fig. 1B). Despite the clear evidence of elevated metals in 35207, only Mn (in all 12 samples) and As (in one 35207 sample) concentrations exceeded EPA Residential Soil Regional Screening Level (RSL) guidelines for human health concerns (As, 0.68; Cd, 7.1; Pb, 400; Mn, 180; and Zn, 2,300 ppm) (27, 28).

**FIG 2:**
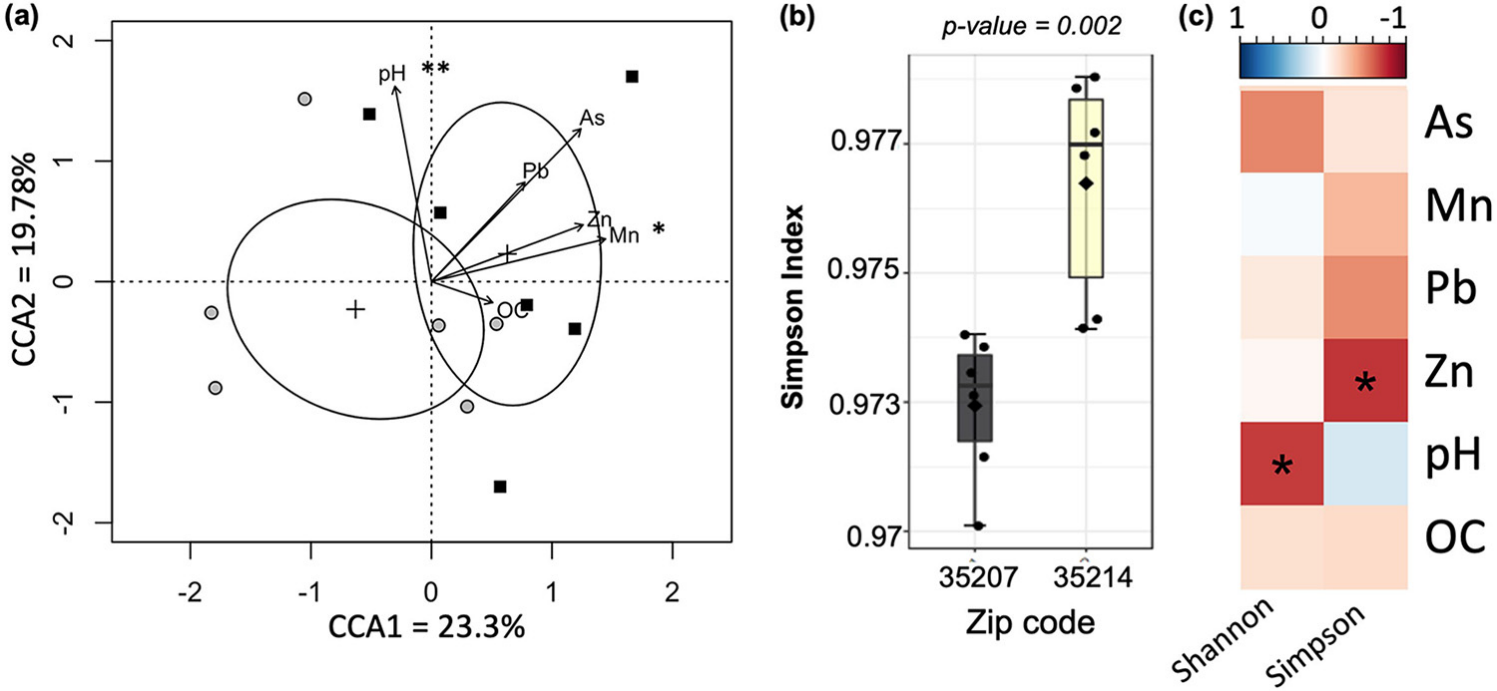
(a) Multivariate canonical correspondence analysis (CCA) showing the first two ordination axes constrained by HM and organic carbon concentrations as well as pH. Ellipses represent 95% confidence intervals of the centroid of the groups, and the vectors indicate the impact of metal concentrations and other environmental variables on the position of points in the plot. Black squares, 35207 samples; gray circles, 35214 samples. (b) Alpha diversity of the bacterial community at the genus level measured by the Simpson Index; the *P* value is from a Mann-Whitney test comparing the two zip codes. (c) Spearman correlation between metal concentrations and the indicated alpha diversity metrics; asterisks indicate that the *P* value of the correlation coefficient is <0.05.

Both nonmetric multidimensional scaling (NMDS) and canonical correspondence analysis (CCA) ordination techniques revealed significant correlations of Mn concentration and pH on soil community structure (Fig. 2A; see S1A in the supplemental material; NMDS ordination also showed a significant effect of Zn). NMDS ordination did not show a significant clustering of samples by zip code (Fig. S1A; analysis of molecular variance [AMOVA] *P* > 0.05), but when ordination was constrained using soil metadata (HM concentrations, soil pH, and organic carbon content) using CCA, the zip codes were clearly differentiated (Fig. 2A). While pH was a significant structuring force, only metal concentrations were important for separating the zip codes, with all four metal biplot vectors indicating movement toward the upper-right corner of the plot (Fig. 2A).

There was also evidence of a negative impact of metals on soil bacterial diversity. The Simpson alpha diversity index, calculated based on OTUs clustered phylogenetically at the genus level, was significantly different between zip codes (Fig. 2B). However, the Shannon index calculated the same way did not show a significant difference, nor did either metric calculated at the OTU level without phylogenetic clustering. Interestingly, both metrics were correlated with metadata; the Shannon index indicated that diversity decreased with increasing pH, and the Simpson index was negatively correlated with Zn concentration (Fig. 2C).

### Impact of metals on bacterial taxonomic groups.

**FIG 3:**
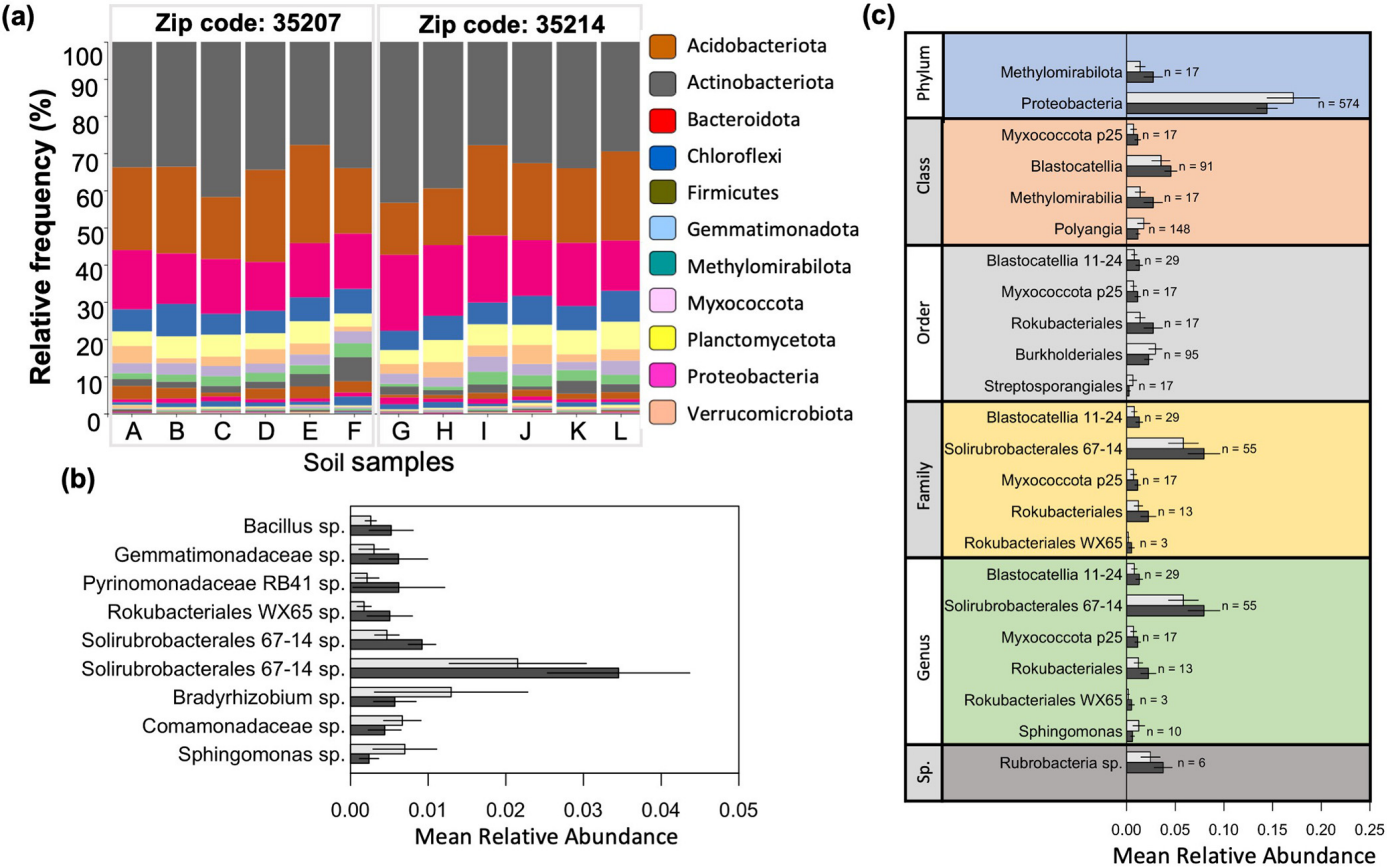
(a) Relative abundance of bacterial phyla in soil bacterial communities based on 16s rRNA sequencing. Sample codes correspond to the map in Fig. 1. (b and c) Mann-Whitney statistical tests were used to estimate significant differences in relative abundances of OTUs (b) or higher-level taxa (c) between 35207 (dark gray bars) and 35214 (light gray bars). For all three plots, only taxa that were at least 1% of at least one community are shown. In panel c, *n* is the number of total OTUs represented by the indicated higher-level taxon.

Across all samples, the most abundant bacterial phyla were *Actinobacteriota*, *Acidobacteriota*, *Proteobacteria*, and *Chloroflexi*, representing 73% to 83% of total bacteria (Fig. 3A). Of the 48 OTUs that reached a relative abundance of at least 1% in at least 1 sample, 9 were significantly different between sites (Fig. 3B). Of these 9, 6 were more abundant in 35207, including the most abundant of the 9, a representative of the Gram-positive *Solirubrobacterales* 67-14 clade. Of higher-level taxa composing at least 1% of one sample, two phyla, four classes, five orders, five families, six genera, and one species were significantly different between sites (Fig. 3C). Notably, the highly abundant phylum *Proteobacteria* was significantly less abundant in 35207, whereas *Methylomirabilota*, a poorly studied group containing the as-yet uncultivated *Rokubacteriales*, was more abundant in 35207. While the phyla *Acidobacteriota* and *Actinobacteriota* were not overall different between zip codes, specific subgroups (e.g., the *Blastocatellia* and *Solirubrobacterales*, both more abundant in 35207) were.

Several taxa were significantly correlated with environmental parameters measured at the sampling sites, including metals (Fig. S2 to S4). Higher organic carbon content was correlated with higher ratios of archaea to eubacteria, driven by the abundance of operational taxonomic units (OTUs) similar to the ammonia-oxidizing archaeal genus *Nitrososphaera*. Lower pH was correlated with greater representation of bacteria from the phylum *Planctomycetota*. Two classes within the phylum *Chloroflexi* were also correlated with pH, with the *Chloroflexia* favoring low pH and the KD4-96 clade favoring higher pH. Unsurprisingly, the *Acidobacteriota* family *Vicinamibacteraceae* was correlated with lower pH.

At the phylum level, only the *Verrucomicrobiota* were correlated with metal concentrations, being rarer in higher-Mn samples (Fig. S2). The class *Polyangia* (*Myxococcota*) as well as the orders *Thermoanaerobaculales* (*Acidobacteriota*) and *Streptosporangiales* (*Actinobacteriota*) were negatively correlated with Zn, and *Tepidisphaerales* (*Planctomycetota*) was negatively correlated with both Zn and Pb (Fig. S3). Other taxa were positively correlated with metals: the *Myxococcota* bacteriap25 class (recently reclassified as members of candidate phylum *Binatota* [29]) was more abundant in higher-Zn samples, the *Solirubrobacterales* 67-14 clade was positively associated with both Pb and Zn, and the *Rokubacterales* WX65 genus was positively correlated with both Mn and Zn. No significant correlations were observed between any taxon and As (Fig. S3).

Interestingly, of the 48 OTUs that composed at least 1% of at least 1 sample, no significantly negative correlations with metal concentrations were observed, whereas 11, or 23%, were positively correlated with at least one metal (Fig. S4). Of these, five were positively associated with two metals, and three with all three metals, Mn, Pb, and Zn. Six of these OTUs were identified as significantly different between zip codes by both Metastats and linear discriminant analysis effect size (LEfSe) analyses; of these, five were positively correlated with at least two metals. Of the 48, 16 (33%) were also significantly correlated with at least 1 of the first 2 NMDS axes (Fig. S1B), with 3 biplot vectors (*Pyrinomonadaceae* RB41, *Rubrobacteria* spp., and *Gemmatimonadaceae* spp., all also positively correlated with metals), pointing to the same quadrant as the metal biplot vectors.

### Influence of metals on predicted soil metagenomes.

**FIG 4:**
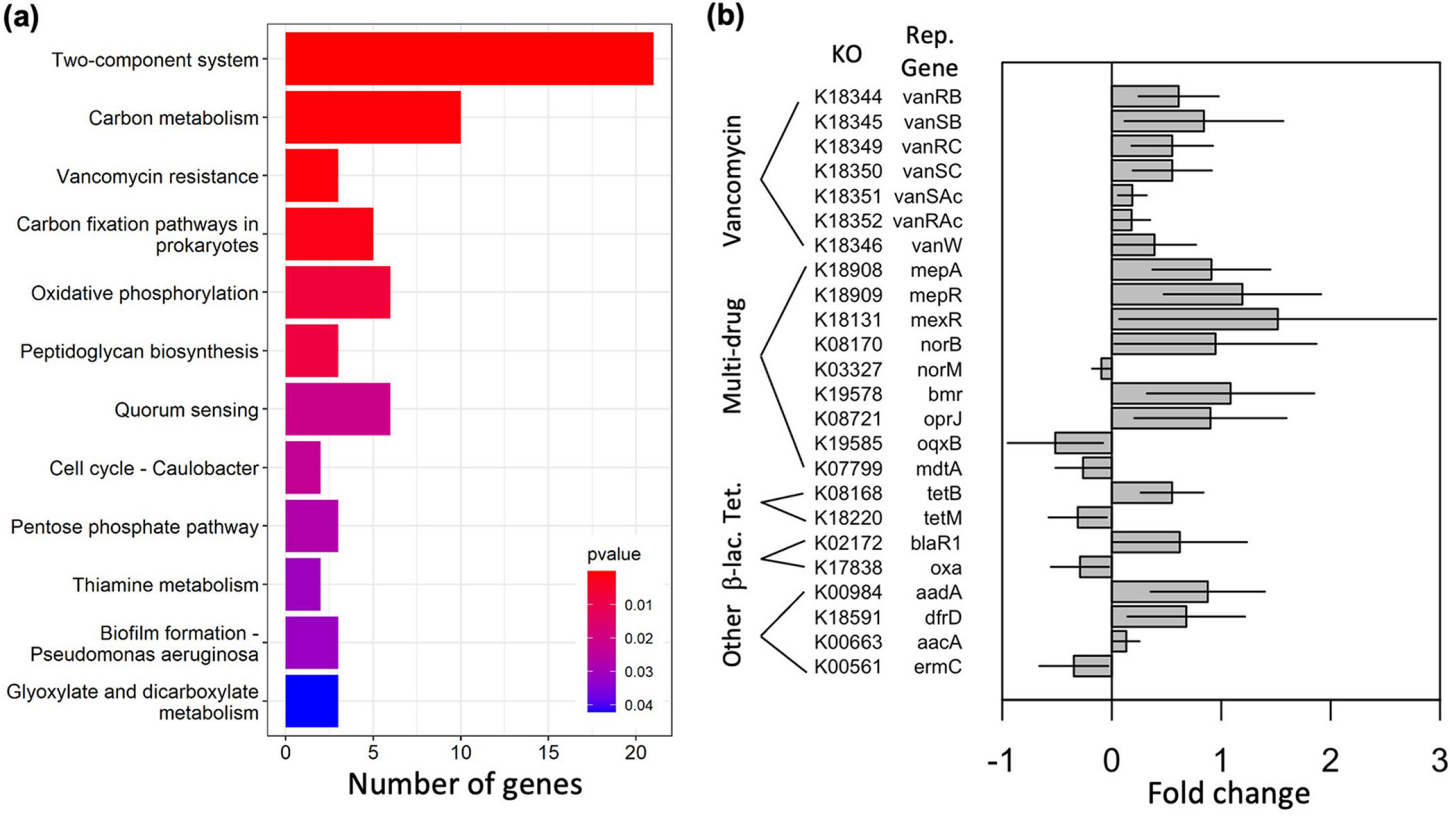
(a) Overrepresentation analysis of differences between gene presence/absence in 35207 versus 35214 based on PICRUSt metagenome inference. Bars indicate the number of genes found to significantly differ between the zip codes that fall into the indicated pathway; *P* values indicate the probability of finding that many differentially represented genes by chance. (b) Antimicrobial genes that differ significantly between zip codes based on PICRUSt metagenome inference. Positive values indicate enrichment in 35207; negative values indicate enrichment in 35214. Error bars represent the 95% confidence interval of the estimate.

We used PICRUSt2 to infer the metagenomes of our 12 soil samples based on 16S profiles, predicting 7,637 unique KEGG Orthology (KO) IDs which allowed us to predict the functional potential of the microbial communities. The functional profiles of the two zip codes were structured significantly differently (NMDS on Bray-Curtis distance, nonoverlapping 95% confidence intervals of centroids, Fig. S5A), with much greater dispersion in the ordination coordinates of the 35214 samples than of those from 35207 (areas of the 95% confidence interval ellipsoids, 0.011 and 0.003, respectively). The same general conclusions held when we constrained the ordination to just the AMR and HMR genes in our predictions (Fig. S5B, ellipsoid areas of 35214 and 35207 are 0.013 and 0.006, respectively). We found that genes from two-component sensory systems, carbon fixation and catabolism pathways, and vancomycin resistance were most likely to differ between the zip codes (Fig. 4a).

We assessed the difference between predicted gene abundances in the two zip codes using Welch’s unequal variance *t* test in STAMP (30). The 2-group tests estimated a significant difference in the mean proportion effect size of 377 genes out of 7,637 between the 2 zip codes (adjusted *P* value < 5%, Table S6). Of these genes, 24 (6.4%) were associated with AMR pathways, and 9 were associated with HMR (Fig. 4B, Table S9). Overall, 46.2% of the significantly differently abundant genes were more abundant in 35207 samples, compared to 75% of differently abundant AMR genes; AMR genes were thus significantly more likely to be different between the sites based on Fisher’s exact test (*P* = 0.005). All seven identified genes related to vancomycin resistance, as well as six of nine genes identified as being involved in multidrug resistance, had higher relative abundance in 35207. Interestingly, only five of nine genes related to HMR were significantly more abundant in 35207, and HMR genes were not more likely to be significantly different between zip codes than other types of genes (Fisher’s exact test, *P* = 0.41).

We also observed direct correlations between metal concentrations and the abundances of predicted AMR and HMR genes. Altogether, Pb, Mn, and/or Zn were significant predictors of 21 AMR/HMR gene abundances (linear model, *P* < 0.05 for the slope of metal concentration versus abundance being not equal to 0; Table S10). Of the genes significantly impacted by Pb, 100% were more abundant at higher Pb concentrations, and only 1 of the 12 genes impacted by Mn was less abundant at higher Mn concentrations (Table 1). Zn, on the other hand, was negatively related to AMR/HMR gene abundance in 5 of 6 significant interactions, and in all 5 cases, genes that were negatively related to Zn concentration were positively related to Pb concentration.

**TABLE 1:** AMR and HMR genes predicted by metal concentrations^*a*^

Class	KO	Gene	Annotation	Pb	Mn	Zn
AMR	K00561	*ermC*, *ermA*	23S rRNA (adenine-N6)-dimethyltransferase (EC 2.1.1.184)	+		−
AMR	K08217	*mef*	MFS transporter, DHA3 family, macrolide efflux protein	+		−
AMR	K18220	*tetM*, *tetO*	Ribosomal protection tetracycline resistance protein	+		−
AMR	K08167	*smvA*, *qacA*, *lfrA*	MFS transporter, DHA2 family, multidrug resistance protein	+		
AMR	K19062	*arr*	Rifampin ADP-ribosylating transferase	+		
AMR	K18909	*mepR*	MarR family transcriptional regulator, repressor for *mepA*		+	+
AMR	K18131	*mexR*	MarR family transcriptional regulator, repressor of the *mexAB*-*oprM* multidrug resistance operon		+	
AMR	K18589	*dfrA1*, *dhfr*	Dihydrofolate reductase (trimethoprim resistance protein) (EC 1.5.1.3)		+	
AMR	K18780	*bla*_NDM_	Metallo-beta-lactamase class B NDM (EC 3.5.2.6)		+	
AMR	K18792	*bla*_OXA-10_	beta-Lactamase class D OXA-10 (EC 3.5.2.6)		+	
AMR	K18824	*sul2*	Dihydropteroate synthase type 2 (EC 2.5.1.15)		+	
AMR	K19096	*bla*_CMY-2_	beta-Lactamase class C CMY-2 (EC 3.5.2.6)		+	
AMR	K19274	*aph3-VI*	Aminoglycoside 3′-phosphotransferase VI (EC 2.7.1.95)		+	
AMR	K19278	*aac6-Ib*	Aminoglycoside 6′-*N*-acetyltransferase Ib (EC 2.3.1.82)		+	
AMR	K19276	*aac3-IV*	Aminoglycoside 3-*N*-acetyltransferase IV (EC 2.3.1.81)		−	
Metal	K07156	*copC*, *pcoC*	Copper resistance protein C	+		−
Metal	K07241	*nixA*	High-affinity nickel-transport protein	+		−
Metal	K07665	*cusR*, *copR*, *silR*	Two-component system, OmpR family, copper resistance phosphate regulon response regulator CusR	+		
Metal	K07230	*p19*, *ftrA*	Periplasmic iron binding protein	+		
Metal	K07311	*ynfG*	Tat-targeted selenate reductase subunit YnfG		+	
Metal	K07490	*feoC*	Ferrous iron transport protein C		+	

a +, positive slope of gene abundance versus metal concentration; −, negative slope.

### Cross-resistance to antimicrobials in HMR bacterial isolates.

**FIG 5:**
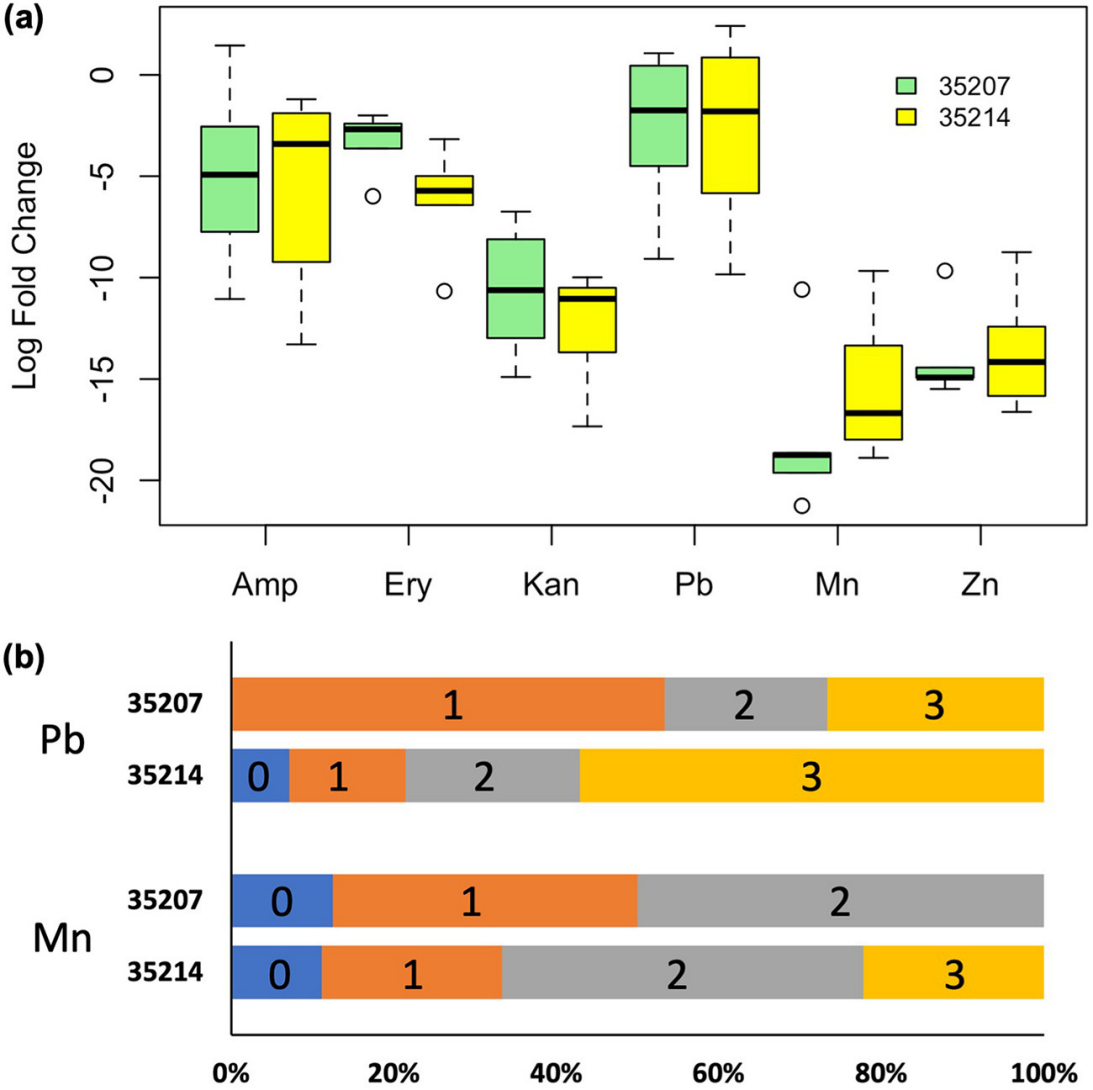
(a) Recovery of colonies on antibiotic- and heavy metal-treated PYT80 plates from soil samples taken from zip codes 35207 and 35214, shown in green or yellow boxes, respectively. Values indicate the base 2 logarithm of the ratio of growth on metal- or antibiotic-treated plates to growth on unamended plates. Bars represent the interquartile range, with the central bar representing the median value and the whiskers extending to the most extreme points equal to or less than 1.5 times the interquartile range from the median, with circles representing outliers beyond this range. (b) Bars indicate the proportion of bacterial isolates taken from plates containing the indicated metal that were also resistant to the indicated number of the 3 tested antibiotics (ampicillin, erythromycin, and kanamycin).

We tested the ability of bacteria from each sample to grow on media containing metals or antibiotics. There were no significant differences in AMR or HMR between samples in 35214 and 35207 when intact soil communities were diluted onto PYT80 agar plates (Wilcoxon tests, *P* > 0.05; Fig. 5A), nor were samples from sites with higher metal concentrations significantly more AMR or HMR (linear models, *P* > 0.05). Except for Pb (in both zip codes) and ampicillin (in 35207), all additions significantly reduced bacterial growth relative to the unamended control plates (Wilcoxon signed rank tests, *P* < 0.05).

To assess the resistance phenotypes of individual strains from each zip code, we isolated 46 strains across zip codes that were able to grow on PYT80 plates spiked with Mn (17 isolates) or Pb (29 isolates); no isolates were obtained from Zn-spiked plates. Based on 16S rRNA sequences, 24 taxonomically distinct isolates were identified based on their closest match in the BLAST nonredundant (nr) database (Fig. S6). The bacterial isolates were mostly *Actinobacteriota* and *Proteobacteria* and were not broadly representative of the taxonomic diversity revealed by our 16S tag sequencing efforts (Fig. S6). Of the isolated taxa, 12 were unique to 35207, 8 were unique to 35214, and 4 were common across both zip codes. The cross-tolerance of the 46 strains was tested by inoculating axenic cultures onto PYT80 plates supplemented with either ampicillin, kanamycin, or erythromycin. In contrast to the broad inhibition of the bulk communities by antibiotics, 94% of HMR isolates were resistant to at least one antibiotic, and 30% were resistant to all three antibiotics used (Fig. 5B). There was no significant difference between zip codes or the metal spike used during strain isolation in the number of antibiotics a strain was resistant to (linear mixed-effects model with phylogenetic identification as the random effect, *P* > 0.05 for each fixed-effect predictor) or in the likelihood that a strain was specifically resistant to ampicillin or kanamycin (binomial mixed-effects regression, *P* > 0.05 for each predictor). Strains isolated from 35214 were, however, significantly more likely to be resistant to erythromycin (binomial mixed-effects regression, *P* = 0.02). For taxa that were isolated multiple times, there was substantial variation between strains in the antibiotic resistance profile (Table S8). For instance, out of nine Rhodococcus degradans isolates, six were resistant to all antibiotics, two were susceptible to erythromycin, and one was susceptible to all three antibiotics tested.

## DISCUSSION

The 35th Avenue Superfund Site in North Birmingham, Alabama, houses coke and coal industries that have left a legacy of HM pollution behind, increasing the risk of lung and other diseases for residents (4, 7). Ongoing efforts to understand and mitigate the human impacts of this environmental injustice have generally looked to the direct effects of HMs on human biology, but to our knowledge, there is little work exploring the possible relationship between HM contamination and AMR prevalence at any U.S. Superfund site. However, several recent studies have found that HM pollution can select for environmental AMR (6, 14, 19, 31–33), so understanding how HM contamination affects microbial diversity is an important step toward achieving environmental justice for residents impacted by the 35th Avenue Superfund Site (4).

First, this study showed a difference between soil bacterial communities from the 35th Avenue Superfund site (zip code 35207) and those in a less polluted nearby neighborhood (zip code 35214). Concentrations of HMs As, Pb, Mn, and Zn were elevated in 35207 compared to 35214, and kmer clustering confirmed that the zip codes could be distinguished by their metal profiles (Fig. 1B). While only Mn and As surpassed EPA guidelines determining unacceptable levels of residential soil pollution, HMs nevertheless had a significant effect on microbial community composition (Fig. 2A, Fig. S1A), mirroring previous work (34). Metal contamination significantly reduced microbial diversity, with Zn exerting an especially strong impact, possibly indicating overdominance of metal-tolerant species in Superfund site soils (Fig. 2B and C).

The abundances of several bacterial taxa were significantly affected by chronically metal-polluted soils (Fig. 3). Some taxa were significantly different between the zip codes, including highly abundant groups such as the *Proteobacteria* (lower in 35207) and the *Solirubrobacterales* (higher in 35207). Consistent with their overrepresentation in Superfund-affected soils in our study, *Solirubrobacterales* have been previously identified as an indicator taxon in metal-contaminated mining and agricultural areas (35, 36) and to significantly contribute to AMR in soils (34). Organisms from this group have also been found to associate with metal-accumulating plants (37) and to become enriched during experimental metal additions to agricultural soils (38).

Absolute metal concentrations were also significant predictors of taxon abundance, and approximately one in four of the most abundant OTUs in our data set were positively correlated with at least one of the metals Mn, Pb, and/or Zn (Fig. S4). Interestingly, this included several representatives of uncultured candidate taxa (e.g., *Rokubacteriales*, *Binatota*) thought to be important in lithotrophic and methylotrophic nutrient cycling processes as well as alkane degradation (29, 39, 40), which may reflect selection by organic contaminants that tend to be deposited along with heavy metals by coal-burning industries. These uncultured taxa have only recently been named, but at least one study has shown enrichment of *Rokubacteriales* at a heavy metal mining site (41). Overall, the magnitude of the impact of metals on community structure was roughly equivalent to that of pH, which has been shown previously to be the dominant structuring factor for soil microbiomes across many diverse ecosystems (42, 43). These results support the assertion that HMs are an important selective force determining microbial community structure.

This study found evidence supporting our hypothesis that AMR was more common in metal-contaminated soils. Functional gene abundances inferred from 16S tag sequences included AMR genes related to a wide variety of antibiotic targets such as protein synthesis (tetracycline resistance), peptidoglycan synthesis (beta-lactam and vancomycin resistance), and folate synthesis (trimethoprim resistance), nearly all of which were significantly higher in samples from 35207 (Fig. 4B) and/or positively correlated with heavy metal concentration. Both vancomycin and trimethoprim resistance have been previously shown to cooccur with resistance against various HMs (33, 44). Multidrug resistance genes such as efflux pumps were enriched in 35207 samples. Importantly, these genes include many of the most clinically concerning AMR pathways and reinforce the importance of considering the impact of environmental pollution as an additional vector for AMR evolution, along with clinical and agricultural antibiotic use, and may indicate a further way that HM contamination threatens the health of humans living in impacted areas.

The bacterial isolates from soil samples that were selected using HM-spiked agar were also highly likely to be resistant to multiple antibiotics (Fig. 5B). However, we were unable to detect a significant difference in community-scale phenotypic AMR or HMR in soil bacterial communities (Fig. 5A). It is possible that this discrepancy reflects biases in our metagenome inference software (45), as the Nearest Sequence Taxon ID (NSTI) values for our samples indicated relatively low representation of many of our taxa in published databases, although our pipeline removed highly divergent OTUs from the metagenome inference to minimize this problem. A more likely cause is that culture-based assays of soil communities are potentially misleading due to the strong cultivation bias in these systems (46). The great majority of our isolates fell into a few clades that were not closely related to the most abundant taxa from our tag sequencing analysis (Fig. S6), and it is noteworthy that only 3 of the 48 OTUs making up more than 1% of any sample (*Bacillus*, *Rhizobium*, and *Streptomyces*) had a close relative among the isolates.

Our results fall short of demonstrating a stronger connection between HMR and AMR at the 35th Avenue Superfund Site due to methodological limitations. For example, PICRUSt2 accuracy is higher for human microbiome samples than for soils (45), so our prediction results should be cautiously interpreted, but also, even under ideal circumstances, it is likely difficult to infer the presence of highly mobile genes like those involved in AMR merely from the taxonomic information provided by 16S tag sequencing. Future work should target specific HMR or AMR genes of interest using quantitative PCR (20) or plasmidomics (47) or mobilomics (48) to improve detection of resistance genes on mobile genetic elements that may be exchanged between soil species and possibly between those organisms and counterparts in the human microbiome (20, 21). Culture-based assays could also be improved, for instance, by performing 16S tag sequencing of enrichment cultures following exposure of intact communities to selective concentrations of HMs or antibiotics, especially in conjunction with metagenomic sequencing.

**Conclusion.** In conclusion, soil microbial communities at an HM-polluted Superfund site (zip code 35207), compared with those from a neighboring zip code (35214) with less HM exposure, were differentially structured according to their HM concentrations. Moreover, 35207 samples showed differences at the genus level in ecologically important taxa, and a significantly greater abundance of predicted AMR genetic markers was detected in 35207. This work compliments other studies showing a connection between HM contamination and AMR and further shows this connection in a heavily populated urban setting with potential ramifications for the health of the humans who live near the 35th Avenue Superfund site. Future work should incorporate more detailed data sets, especially relating to residential knowledge of land use and edaphic parameters such as soil pH, soil type, plant cover (34, 49, 50), and fungal diversity (11), and ideally should also investigate the microbiomes of the human residents of the zip codes to determine the degree to which HMs impact AMR diagnoses such as recalcitrant infections. Taken together, these results support the continued scientific and legislative advancement of environmental and health justice for residents of the 35th Avenue Superfund Site and highlights the necessity of AMR stewardship programs for health care policy at Superfund sites.

## MATERIALS AND METHODS

### Site description and soil sampling.

This project began as a set of course-based undergraduate research experiences using methods as previously described (51). On 14 September 2019, three soil samples each were collected at six different public access parks in the North Birmingham, Alabama, 35207 zip code, for a total of 18 soil samples. Each sample (5 g) was collected from the topsoil layer with an ethanol-sanitized trowel and a sterile plastic bag. The three sampling points at each site were chosen based on ease of access instead of using a randomized grid design; however, they were well separated and at leas 1 m from any large vegetation such as trees or shrubs. Sampling was repeated on 15 September in the 35214 zip code for 18 additional soil samples. A small amount of precipitation fell on 14 September (<0.5 inches in the morning prior to sampling), and no precipitation occurred on 15 September. According to the National Weather Service (weather.gov), the last significant precipitation prior to sampling was 27 August. All three samples per park were then homogenized by vigorous shaking, such that each park’s final sample contained three mixed replicates with a final yield of ~15 g. Overall, we collected a total of 12 homogenized soil samples, six from 35207, and six from 35214 (Fig. 1A). These 12 park sites were chosen due to their public accessibility to the community residents. In September 2020, soil sampling was repeated from five of the original six parks per zip code to provide fresh soil samples for culture assays. One public park per zip code was not resampled due to access limitations during the COVID-19 pandemic. All soil samples to be used for nucleic acid purification and sequencing were immediately stored at −80°C until further processing.

### Heavy metal, organic mass, and pH testing.

Soil (5 to 6 g from each 2019 sample) was tested for heavy metal concentrations by Sutherland Environmental Testing using EPA Method 6010B (EPA laboratory ID AL01084) with inductively coupled plasma-atomic emission spectroscopy. The analytes reported were As, Cd, Pb, Mn, and Zn. The reported limit of detection was 1 ppm for each analyte. Estimated concentrations below this detection limit were assumed to be 0 for the purposes of our analyses. Kmer clustering of collection sites based on their HM profiles was done using the kmer package (52) in R v1.4.1717 (53). The remaining soil samples (from the 2019 samples) not used for nucleic acid purification and sequencing were stored in the dark at room temperature until pH and organic mass testing. We determined organic mass as the percent weight loss-on-ignition (LOI); portions of soil samples were oven-dried at 100°C for 1 h, weighed, heated in a muffle furnace at 400°C for 3 h, and reweighed. Lastly, pH (from the 2019 samples) was measured via electrode after suspending 1 g of soil in 2.5 mL distilled water.

### Nucleic acid purification and sequencing.

DNA was purified and sequenced from each of the 12 homogenized samples (six from 35207 and six from 35214) from the 2019 collection. Bacterial genomic DNA was extracted from 0.20 g of soil using the Qiagen DNeasy PowerSoil Pro kit (Germantown, MD, USA, catalog [cat.] no./ID 47016) according to the manufacturer’s instructions, including an initial step of beat-beating at 4 m/s for 20 s with a FastPrep-24 instrument (MP Biomedicals). The quality of DNA was confirmed by spectrophotometry using Gen5 software with a Take3 microvolume plate in a BioTek Synergy H1 microplate reader (A260/280, ~1.8; each sample contained at least 65 ng/μL of DNA). At the University of Alabama at Birmingham Heflin Center for Genomics (Birmingham, Alabama), an amplicon library was created via PCR amplification of the hypervariable region 4 (V4) of the 16S rRNA gene using barcoded oligonucleotide primers F515 (CACGGTCGKCGGCGCCATT) and R806 (GGACTACHVGGGTWTCTAAT) (54). Genomic DNA was then gel-purified and sequenced using the Illumina MiSeq platform. The demultiplexed paired-end 16S rRNA sequence reads were submitted to the NCBI SRA database and can be accessed through BioProject accession no. PRJNA828526.

### Microbiome sequence processing and statistical analysis.

DNA sequences were processed with the Quantitative Insights into Microbial Ecology package (QIIME2-2020.11) (55, 56) (Table S1). The demultiplexed paired-end reads obtained from Illumina sequencing were processed with the denoising algorithm DADA2 (57). The minimum and maximum demultiplexed read counts were 163,408 and 359,799 respectively. The sequences were truncated to a length of 250 bp. The representative sequences obtained after denoising were clustered using q2-vsearch (QIIME2 plugin) *de novo* clustering at a 97% similarity threshold to remove singletons and obtain 4,185 unique operational taxonomic units (OTUs). The OTUs were classified using a pretrained naive Bayes classifier on silva-138-99-515-806-nb-classifier, trained earlier on 515F/806R region of sequences from the Silva-138 99% OTU database (QIIME2 Data resources-MD5, e05afad0fe87542704be96ff483824d4 (58, 59); https://docs.qiime2.org/2021.2/data-resources) (Table S2). The q2-feature-classifier plugin was used to taxonomically identify the OTUs and remove nonbacterial OTUs (58). All codes necessary to replicate these and other analyses, along with necessary raw data, can be found at https://doi.org/10.5061/dryad.kkwh70s86.

Alpha diversity (Shannon and Simpson indices) at the genus level was estimated using MicrobiomeAnalyst (https://www.microbiomeanalyst.ca) (60, 61) after applying filters for low-count (<4 counts in more than 20% of samples) and low-variance (limited to the interquartile range) OTUs and normalizing by total sum scaling. For beta diversity analyses, the OTU table was first rarefied to the level of the least deeply sequenced sample using a ranked subsampling algorithm (62). Canonical correspondence analysis (CCA) was completed using the vegan v2.5 package (63) in R. Principal coordinates and nonmetric multidimensional scaling (NMDS) ordination were performed in mothur (64) using distance matrices generated with one of several different metrics; the ordination technique and distance method that gave the best r-squared value on three axes (NMDS with the Yue-Clayton theta metric, r-squared = 0.97, stress = 0.065) was used for further analysis. Statistical significance of the impact of environmental metadata on NMDS ordination was assessed by calculating the Spearman correlation coefficient between each variable and the first two NMDS axes using the corr.axes command in mothur (Table S3). The influence of individual OTUs on NMDS ordination was also determined with corr.axes (Table S2).

Differential abundance of OTUs between zip codes was determined using Metastats (implemented through mothur) and LEfSe (implemented through MicrobiomeAnalyst) (65, 66) (Table S2). Spearman correlations between OTUs (Table S2) and higher taxon abundances (Table S4) and environmental metadata (metals, pH, organic carbon) were calculated in R. Significance levels of differences in OTU abundances between zip codes was computed using a simple two-tailed *t* test for each OTU individually (Table S4). Only OTUs that represented at least 1% of the overall community in at least one sample were considered further for these OTU-level analyses.

### Predictive metagenomic analysis.

16S amplicon data were used to predict microbial population metagenomes using Phylogenetic Investigation of Communities by Reconstruction of Unobserved States (PICRUSt v2.3.0) (45) (Table S6). The 16S rRNA FASTA sequence and abundance biom (Biological Observation Matrix) table was used to predict Kyoto Encyclopedia of Genes and Genomes (KEGG) genes and pathway abundances. Sequences with Nearest Sequence Taxon ID (NSTI) above a cutoff of 2.0 were removed along with their sequence counts (Table S5). The OTU abundances were multiplied by the corresponding NSTI value of each OTU, and weighted NSTIs of each sample were calculated from the sum of the column per sample and divided by the total read depth per sample (0.27 ± 0.02 from 35207 and 0.25 ± 0.035 from 35214). Weighted NSTI values describe the degree to which microbes in samples are related to known genomes, where a value of 0.10 would represent 90% representational similarity. While lower numbers are preferred, NSTI values of 0.17 to 0.28 have been previously reported for microbial populations derived from soil samples and yielded useful representations of metagenomes (45, 67–69). The predicted metagenomes were functionally annotated with PICRUSt2 using the KEGG pathway database (70). The KO IDs obtained were manually annotated using the KEGG database to estimate AMR and HMR gene abundance. KEGG genes were compared between the two zip codes using Welch’s *t* test in STAMP (Statistical Analysis of Taxonomic and Functional Profiles) v2.1.3 (30). The relationships between gene abundances and environmental metadata were computed as gene-by-gene linear models in R, as was Fisher’s exact test to determine if AMR or HMR genes were more likely to be significantly correlated with metals than other genes. Overrepresentation of KEGG pathways between the zip codes was estimated using the command enrichKEGG in clusterProfiler (71) (Table S7).

### Bacterial cultivation, identification, and antimicrobial sensitivity determination.

Bacterial cultures from the 2020 collection were isolated on PYT80 agar containing (per L) 80 mg each of peptone, yeast extract, and tryptone, 1.95 g 2-(N-morpholino)ethanesulfonic acid (MES), 15 g purified agar (USP Grade, MP Biomedicals), and 10 mg cycloheximide and adjusted to pH 6.5 (modified by reference 72). Next, 1 mg of soil from each 2020 sample was suspended in 9 mL 0.085% sterile saline and then serially diluted onto PYT80 agar with or without additional heavy metals [0.5 M Pb(NO_3_)_2_ at a final concentration of 0.4 mM; 1 M MnCl_2_ at a final concentration of 25 mM, or 0.5M ZnCl_2_ at a final concentration of 0.5 mM] or antibiotics (erythromycin at a final concentration of 50 μg/mL, ampicillin at a final concentration of 100 μg/mL, or kanamycin at a final concentration of 25 μg/mL). After inoculation, plates were incubated at 20°C for a minimum of 72 h. The overall community sensitivity to the amendments was calculated as the log fold change of the ratio of CFU/mL of HMR or AMR culturable organisms to total culturable organisms (determined by plate counts on amended versus unamended PYT80 plates, respectively).

We also isolated a total of 46 HMR bacterial strains that could grow on PYT80 supplemented with Mn, Zn, or Pb, including representatives from all 12 sites, and identified them by PCR amplification of the 16S rRNA gene using primers UA1406R (5′-ACGGGCGGTGWGTRCAA-3′) and U341F (5′-CCTACGGGRSGCAGCAG-3′) followed by Sanger sequencing at the UAB Heflin Center for Genomics (Birmingham, Alabama). Sequences were trimmed using MEGA X (v.10.2.4) (73) and identified using the Basic Local Alignment Search Tool (BLAST) 16S/ITS BLASTn function against the nr database (BLAST v2.11.0) (74, 75) (Table S8). Of 46 isolates, 43 were successfully amplified and sequenced, and the resulting trimmed sequences were deposited at GenBank under accession numbers ON502989 to ON503031. HMR isolates were subsequently tested for growth on PYT80 with ampicillin, kanamycin, or erythromycin to determine the prevalence of cross-resistance (76, 77); strains forming visible colonies in the presence of antibiotics within 7 days were considered tolerant.
