# Supplementary material for: Heavy Metal Pollution Impacts Soil Bacterial Community Structure and Antimicrobial Resistance at the Birmingham 35th Avenue Superfund Site
Source: Microbiol Spectr. 2023 Mar 23;11(2):e02426-22. doi: 10.1128/spectrum.02426-22 (PMC10101053; doi:10.1128/spectrum.02426-22)
Supplement: Supplemental file 1 — Supplemental material. Download spectrum.02426-22-s0001.pdf, PDF file, 3.0 MB [file spectrum.02426-22-s0001.pdf]

Heavy metal pollution impacts soil bacterial community structure and antimicrobial resistance at the Birmingham 35th Avenue Superfund Site

Anuradha Goswami*[1], Sarah J. Adkins-Jablonsky*[1,2], Marcelo Malisano Barreto Filho[1], Michelle D. Shilling[1], Alex Dawson[1], Sabrina Heiser[1], Aisha O'Connor[1], Melissa Walker[1], Qutia Roberts[1], and J. Jeffrey Morris[1,3]

*These two first authors contributed equally to this manuscript.

[1] Department of Biology, University of Alabama at Birmingham, Birmingham, AL, USA
[2]Alabama College of Osteopathic Medicine, Dothan, AL, USA
[3] Author for correspondence: evolve@uab.edu; 1300 University Blvd, CH 464, Department of Biology, University of Alabama at Birmingham, Birmingham, AL, 35294

Supplementary Information

Table S1. Illumina sequence read analysis data.

Sample ID	Area Tag	Zip code	Input Reads	Filtered Reads	% Passing	Denoised Reads	Merged Reads	% of input merged	Non-chimeric Reads	% of input non-chimeric
22	A	35207	273339	119938	43.88	112542	88100	32.23	86201	31.54
23	B	35207	359799	162989	45.3	155053	127629	35.47	123616	34.36
24	C	35207	356165	153405	43.07	145764	116674	32.76	111370	31.27
25	D	35207	323069	155562	48.15	148821	122908	38.04	120018	37.15
26	E	35207	353938	189010	53.4	181023	152082	42.97	147389	41.64
27	F	35207	248656	78826	31.7	72997	48593	19.54	47854	19.25
28	G	35214	242633	103345	42.59	96341	72445	29.86	70899	29.22
29	H	35214	179152	76448	42.67	70996	52576	29.35	51794	28.91
30	I	35214	338831	139445	41.15	132680	106931	31.56	104827	30.94
31	J	35214	163408	71657	43.85	66863	51054	31.24	50412	30.85
32	K	35214	197013	81581	41.41	76523	58285	29.58	56820	28.84
33	L	35214	177511	73398	41.35	68068	48590	27.37	48348	27.24

Table S2. OTU analysis of 16S rRNA sequencing data (see .csv file). Includes taxonomies, results of Metastats and LEFSe analyses, and correlational analysis vs. NMDS axes and soil metadata.

Table S3. Correlation of soil sample NMDS coordinates with heavy metal concentrations.

Metal	Axis 1	p-value	Axis 2	p-value	Axis 3	p-value	Length
As	-0.480	0.111	-0.393	0.192	0.393	0.192	0.735
Pb	-0.364	0.228	-0.531	0.075	0.140	0.643	0.659
Mn	-0.035	0.908	-0.622	0.031	0.259	0.391	0.675
Zn	-0.238	0.430	-0.615	0.033	0.329	0.276	0.737
OC	-0.063	0.835	0.063	0.835	0.427	0.157	0.436
pH	-0.790	0.002	0.343	0.256	0.035	0.908	0.862

Table S4. Abundances of higher-level taxa and correlations with heavy metal concentrations (see .csv file).

Table S5. 16S copy number normalization and NSTI value for each OTU for PICRUSt analysis (see .csv file).

Table S6. Normalized expression levels of KOs from PICRUSt analysis, including correlations with metadata, gene category assignments, and Mann-Whitney U-test and Welch's t-test results comparing zip codes (see .csv file).

Table S7. Overrepresentation analysis of KO expression data (see .csv file).

Table S8. Cross-resistance of bacterial isolates (see .csv file). "Original isolation" indicates the metal that was used in the agar from which the bacterium was isolated. "Amp", "Kan", and "Erm" indicate whether the bacteria were also resistant to the antibiotics ampicillin, kanamycin, and/or erythromycin, respectively. "Total" indicates the total number of antibiotics the particular isolate was resistant to.

Table S9. Mean expression levels of KOs involved in heavy metal (HMR) or antibiotic (AMR) resistance (see .csv file).

Table S10. Multiple linear regression of KO expression levels on soil metadata. Each KO was regressed against soil metadata individually. Values given are the regression coefficients (i.e., slopes) for each predictor metadata variable for expression level.

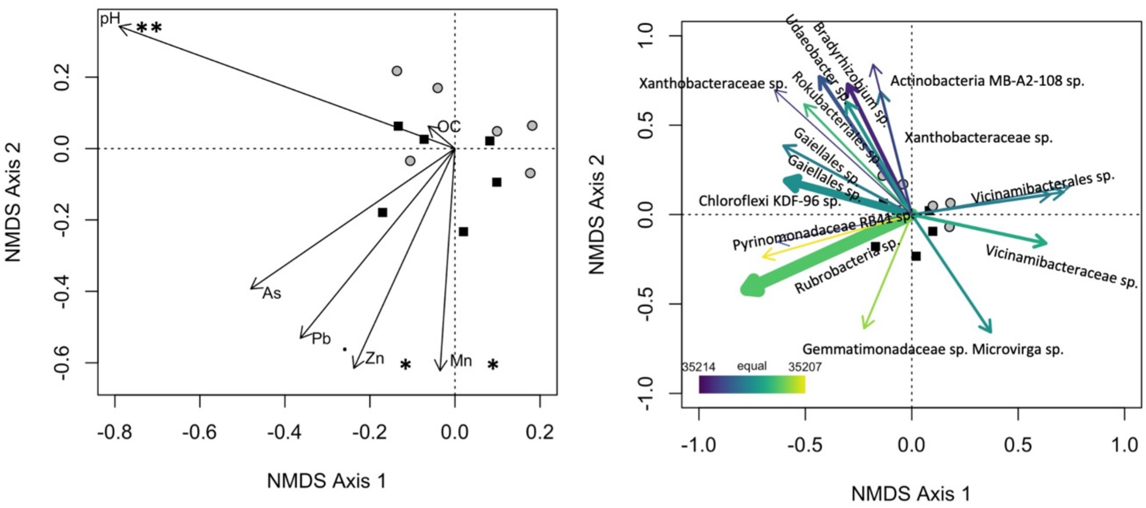

Figure S1. NMDS ordination plots of soil microbial communities. (a) Biplot vectors showing the influence of soil metadata, including metal concentrations, pH and organic carbon (OC) content on position of communities in the plot. Asterisks indicate the significance value of the correlation of the most significant axis with the indicated parameter; *, $p < 0.05$; **, $p < 0.01$. **(b)** The same plot, but showing the biplot vectors of all OTUs that represented at least 1% of any sample. The width of the arrow is proportional to the average relative abundance of the OTU across all samples, and the color indicates the level of bias towards one zip code or the other. Black squares = 35207 samples; gray circles = 35214 samples.

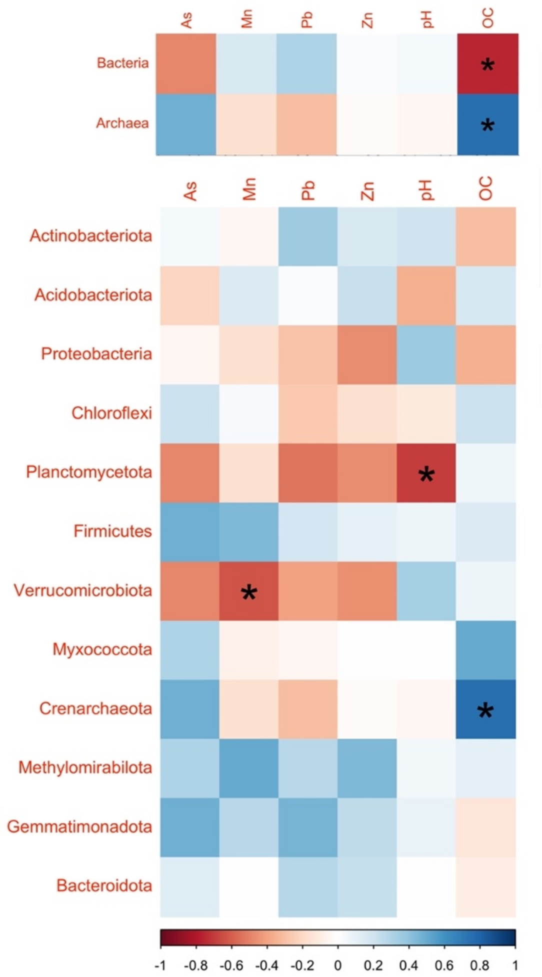

Figure S2. Correlation of Domain and Phylum taxa with soil metadata. Color scale reflects the Spearman correlation coefficient of the taxon's abundance vs. the indicated metadata parameter from each soil sample. *, $p < 0.05$.

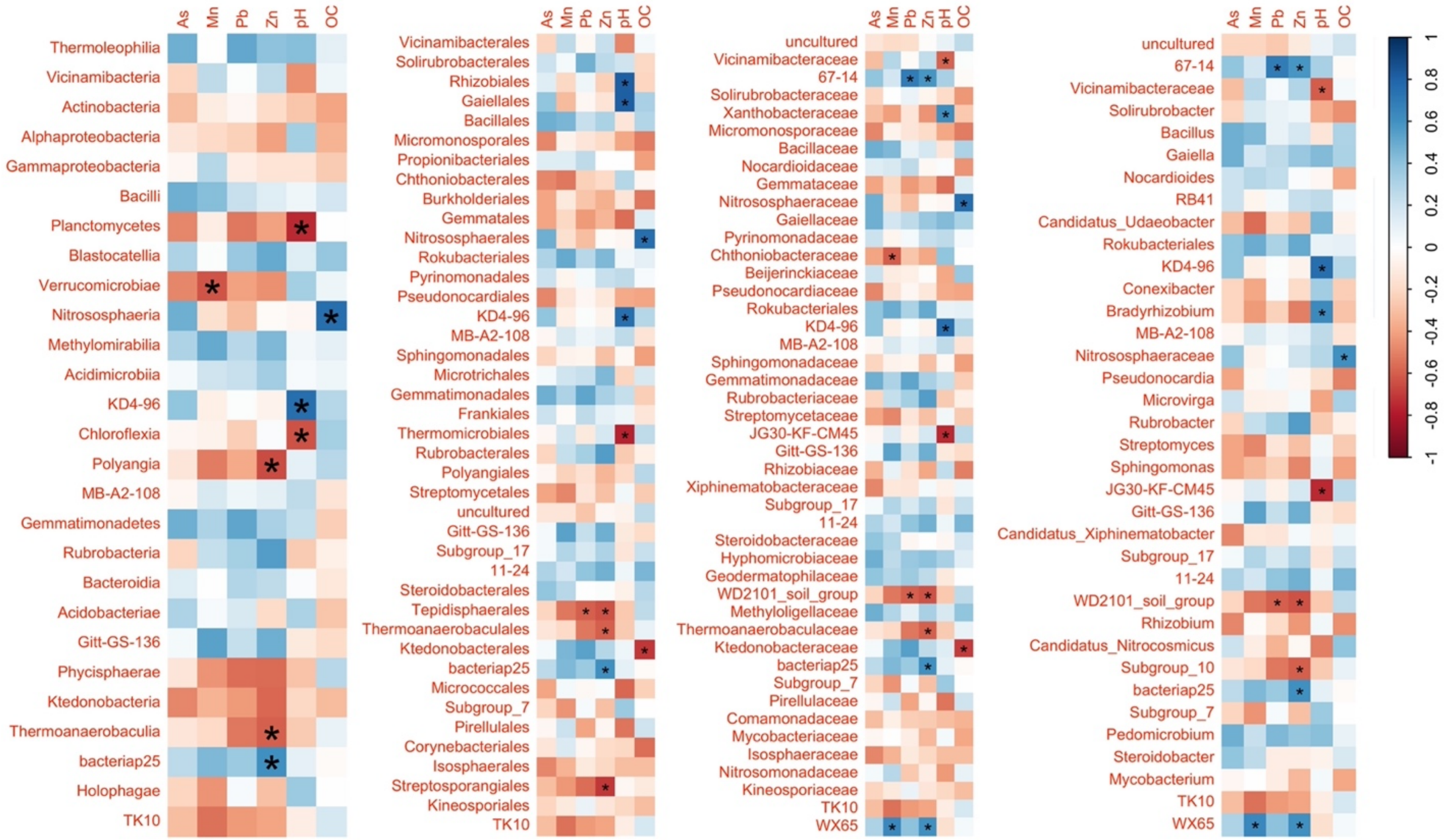

Figure S3. Correlation of Class, Order, Family, and Genus taxa with soil metadata. Color scale reflects the Spearman correlation coefficient of the taxon's abundance vs. the indicated metadata parameter from each soil sample. *, $p < 0.05$.

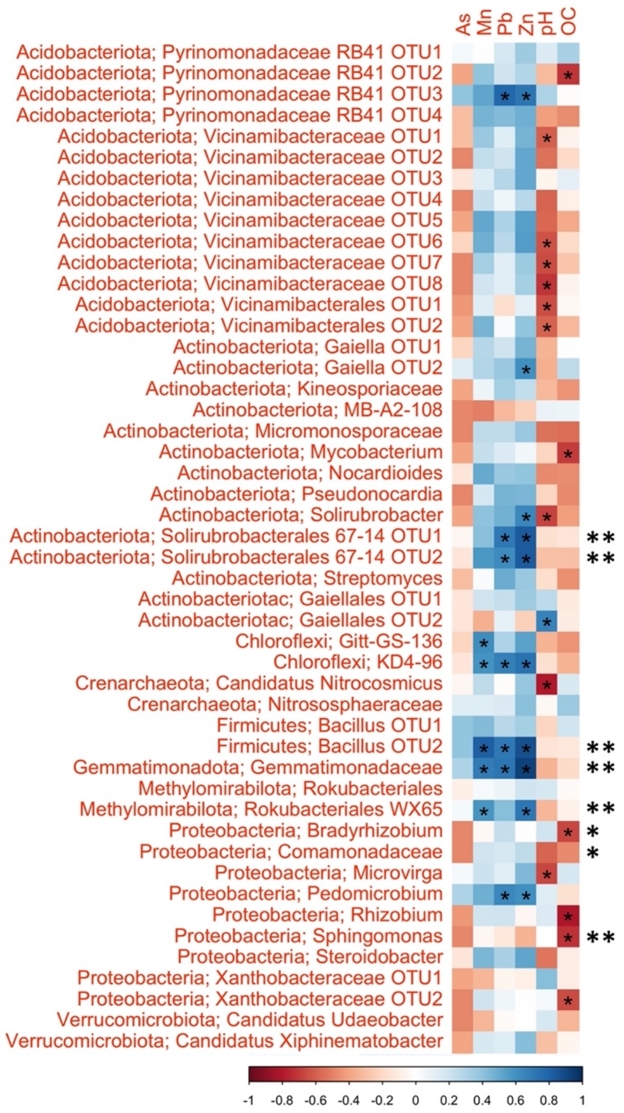

Figure S4. Correlation of OTUs with soil metadata. All OTUs representing at least 1% of any soil sample are shown, along with their taxonomic identifications at the phylum and genus levels. Color scale reflects the Spearman correlation coefficient of the taxon's abundance vs. the indicated metadata parameter from each soil sample. Asterisks within colored squares indicate the significance level of the correlation coefficient, with * indicating $p < 0.05$. Asterisks to the right of the heat map indicate taxa that returned significant adjusted p-values in Metastats (one asterisk) and/or LEFSe (two asterisks) analyses.

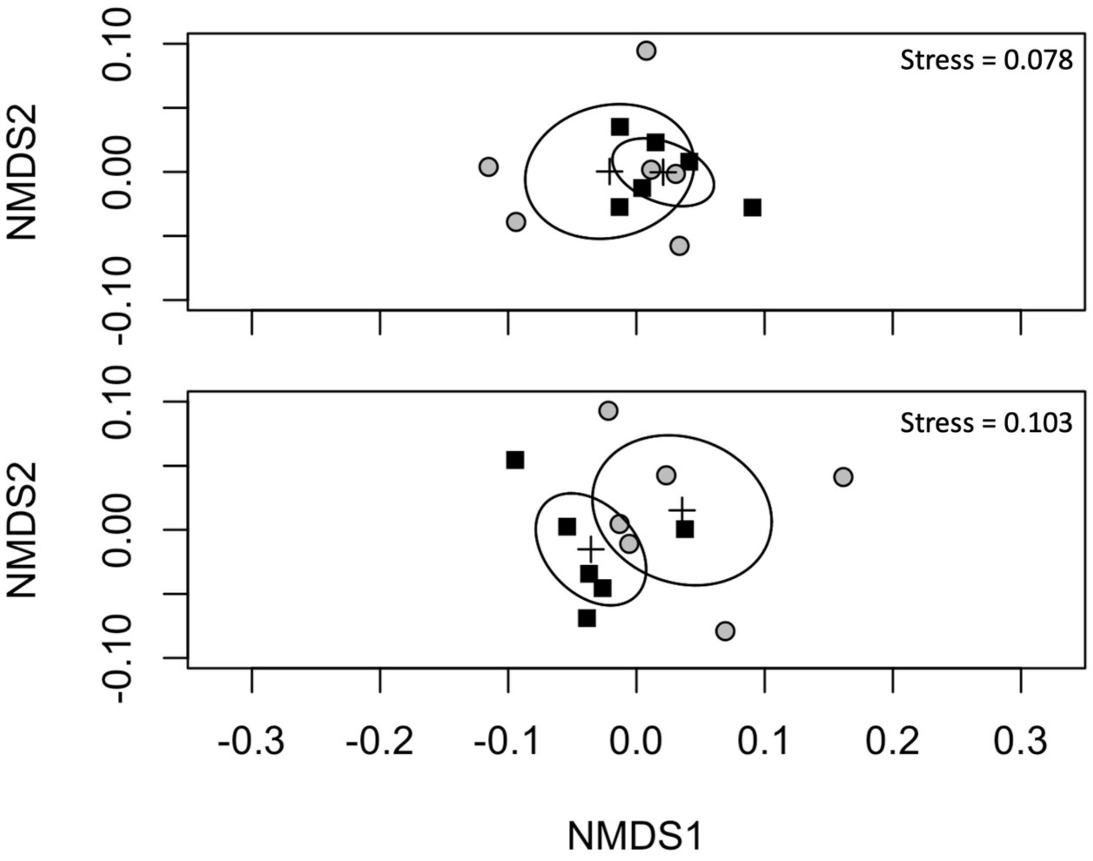

Figure S5. NMDS ordination plots of inferred gene abundance from PICRUSt analysis. The ordination in the top plot used all predicted gene abundances, whereas the bottom plot was restricted to AMR and HMR genes. Crosses and ellipses indicate the centroids and 95% confidence intervals of the zip codes. Black squares = 35207 samples; gray circles = 35214 samples.

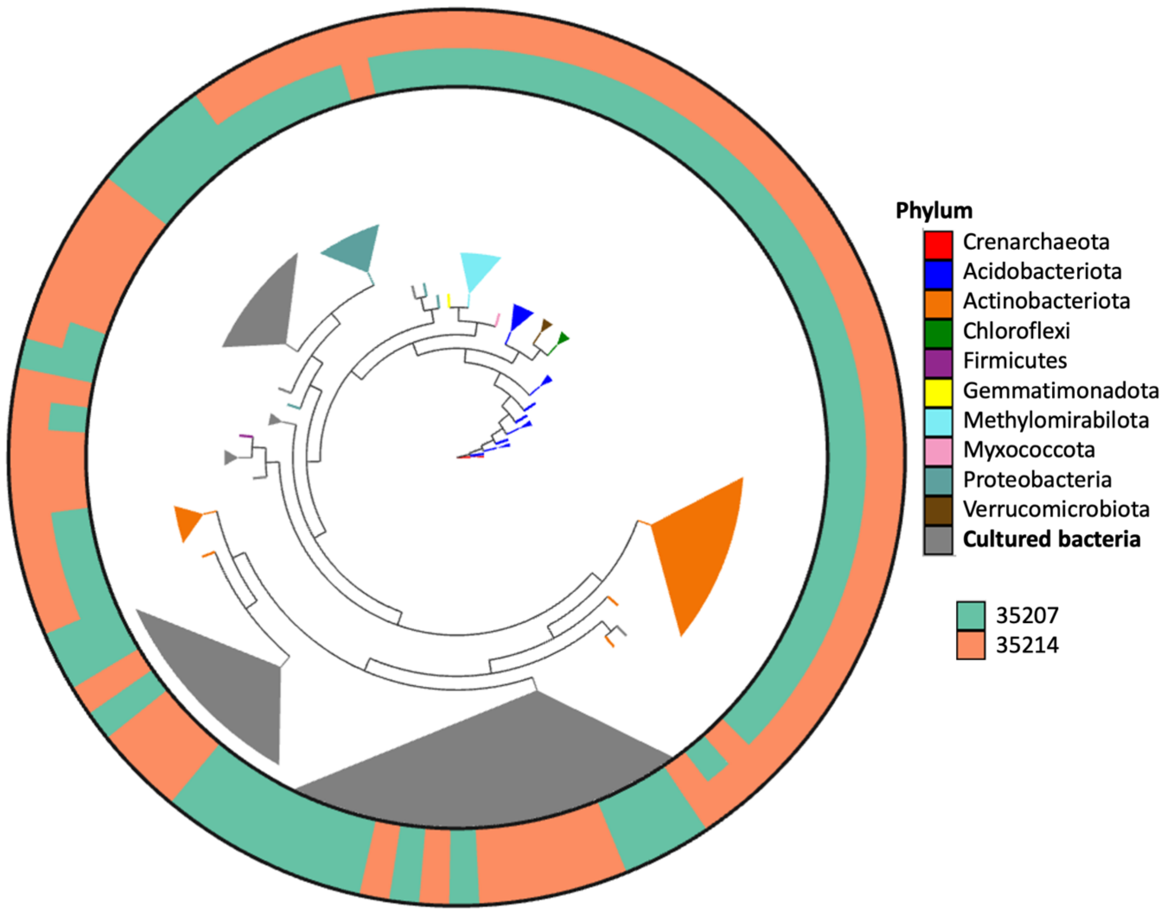

Figure S6. Phylogenetic tree of most abundant OTUs and cultured isolates used in HMR and AMR assays. The phylogenetic tree was assembled using the 16S rRNA sequences of all OTUs representing at least 1% of any sample as well as the 16S samples of all isolates used in our culture-based assays. Colored wedges represent multiple sequences with the same phylum-level taxonomic assignment within the same clade. Colored outer ring indicates whether each taxon was found in 35207, 35214, or both.
